# The potential of retinoic acid receptors as prognostic biomarkers and therapeutic targets in gastric cancer

**DOI:** 10.3389/fonc.2024.1453934

**Published:** 2024-09-11

**Authors:** Silvio Ken Garattini, Debora Basile, Valli’ De Re, Giulia Brisotto, Gianmaria Miolo, Vincenzo Canzonieri, Giuseppe Aprile, Carla Corvaja, Silvia Buriolla, Enrico Garattini, Fabio Puglisi

**Affiliations:** ^1^ Department of Oncology, ASUFC University Hospital, Udine, Italy; ^2^ Department of Medical Oncology, San Giovanni di Dio Hospital, Crotone, Italy; ^3^ Immunopathology and Cancer Biomarkers/Bio-Proteomics Facility, Centro di Riferimento Oncologico, IRCCS, Aviano, Italy; ^4^ Department of Medical Oncology, Centro di Riferimento Oncologico di Aviano (CRO) IRCCS, Aviano, Italy; ^5^ Pathology Unit, IRCCS CRO National Cancer Institute, Aviano, Italy; ^6^ Department of Medical, Surgical and Health Sciences, University of Trieste, Trieste, Italy; ^7^ Department of Oncology, University and General Hospital, Udine, Italy; ^8^ Department of Oncology, San Bortolo General Hospital, Vicenza, Italy; ^9^ Division of Thoracic Oncology, European Institute of Oncology (IEO) IRCCS, Milano, Italy; ^10^ Department of Biochemistry and Molecular Pharmacology, IRCCS-Istituto di Ricerche Farmacologiche “Mario Negri”, Milan, Italy; ^11^ Departiment of Medicine, University of Udine, Udine, Italy

**Keywords:** retrospective-clinical-study, gastric cancer, retinoic-acid-receptors, prognosticbiomarkers, therapeutic-targets

## Abstract

**Background:**

Gastric cancer is a heterogeneous collection of tumors characterized by low survival rates. All-trans retinoic acid (retinoic-acid) is a clinically useful therapeutic agent belonging to the chemical family of retinoids, which consists of both natural and synthetic derivatives of vitamin-A. Retinoids are essential components of the normal diet and they regulate different physiological processes. From a therapeutic point of view, retinoic-acid is the first example of clinically useful differentiating agent. Indeed, the differentiating properties of this compound have promoted the use of retinoic-acid as a standard of care in Acute-Promyelocytic-Leukemia, a rare form of acute myeloid leukemia. In this study, we determine the RNA expression of the six isoforms of *Retinoic*-*Acid*-*Receptors* (*RARα*/*RARβ*/*RARγ*/*RXRα*/*RXRβ*/*RXRγ*) in view of their potential use as gastric cancer progression markers and/or therapeutic targets. In addition, we evaluate associations between the expression of these receptors and a simplified molecular classification of stomach tumors as well as the clinical characteristics of the cohort of patients analyzed. Finally, we define the prognostic value of the various *Retinoic*-*Acid*-*Receptors* in gastric cancer.

**Methods:**

In this single institution and retrospective *RAR-GASTRIC* study, we consider 55 consecutive gastric cancer patients. We extract total RNA from the pathological specimens and we perform a *NanoString Assay* using a customized panel of genes. This allows us to determine the expression levels of the *RAR* and *RXR* mRNAs as well as other transcripts of interest.

**Results:**

Our data demonstrate ubiquitous expression of the *RAR* and *RXR* mRNAs in gastric cancers. High levels of *RARα*, *RARβ*, *RXRα* and *RXRβ* show a significant association with stage IV tumors, “*de novo*” metastatic disease, microsatellite-stable-status, epithelial-to-mesenchymal-transition, as well as *PIK3CA* and *TP53* expression. Finally, we observe a worse *overall*-*survival* in gastric cancer patients characterized by high *RARα*/*RARβ*/*RARγ*/*RXRβ* mRNA levels.

**Conclusions:**

In gastric cancer, high expression levels of *RARα*/*RARβ*/*RARγ*/*RXRβ* transcripts are associated with poor clinical and molecular characteristics as well as with reduced *overall*-*survival*. Our data are consistent with the idea that *RARα*, *RARβ*, *RARγ* and *RXRβ* represent potential prognostic markers and therapeutic targets of gastric cancer.

## Introduction

*Gastric cancer* is a heterogeneous type of tumor and it represents the 5^th^ most common cause of cancer worldwide as well as the 3^rd^ cause of cancer related death (https://www.iarc.who.int/news-events/latest-world-cancer-statistics-globocan-2012-estimated-cancer-incidence-mortality-and-prevalence-worldwide-in-2012/). Despite many improvements in the field of medical oncology, the survival rate of the various forms of *gastric cancer* remains low even following completion of cornerstone clinical trials (1, 2). Thus, an effective and personalized treatment of *gastric cancer* requires the development of novel therapeutic strategies based on a refinement of the available molecular classifications of stomach tumors. The scientific literature presents with two distinct molecular classifications of *gastric cancer*, which rely on the information available in the *TCGA* (*The-Cancer-Genome-Atlas*) (3) and *ACRG* (*Asian-Cancer-Research-Group*) websites (4). From these two classifications, some molecular subgroups have emerged as potentially responsive to specific treatments: *MicroSatellite-Instability* [*MSI*] and *Epstein-Barr-Virus*-[*EBV*]-positive tumors to immunotherapy; *Chromosomal-Instability* [*CIN*] tumors to targeted therapy (5). Although, these two classifications are of little interest in the clinical practice, because of the low reproducibility and elevated costs of the methodologies necessary to generate them. Although, these two classifications are of little interest in the clinical practice, because of the low reproducibility and elevated costs of the methodologies necessary to generate them, they may acquire interest in the coming era of precision oncology. Indeed, routine exome sequencing and *Molecular Tumor Boards* (*MTB*s) are likely to play an increasingly important role in optimizing treatment selection. In addition, routine exome sequencing and *MTB*s are expected to improve outcomes by reviewing and interpreting molecular-profiling data and matching patients with appropriate molecularly targeted therapies.

All-trans retinoic acid (*ATRA*) is a clinically useful therapeutic agent belonging to the chemical family of retinoids, which consists of both natural and synthetic derivatives of vitamin-A (retinol). Retinoids are essential components of the normal diet and they regulate different physiological processes, such as the embryonal development, the reproduction and maturation of cells found in regenerative tissues and the process of vision (6, 7). From a therapeutic point of view, *ATRA* is the first example of clinically useful differentiating agent. Indeed, the differentiating properties of this compound have promoted the use of *ATRA* as a standard of care in *Acute-Promyelocytic-Leukemia*, a rare form of acute myeloid leukemia (8, 9). In addition, *ATRA* is used in the clinical management of neuroblastoma (10). The exceptional clinical results obtained in *Acute-Promyelocytic-Leukemia* and neuroblastoma have raised interest in the use of *ATRA* for the prevention of pre-cancerous lesions and the treatment of solid tumors. With respect to this, it is important to mention that a number of available studies demonstrates the efficacy of *ATRA* in preventing and treating solid tumors such as leukoplakia, actinic keratosis and cervical dysplasia (11–13), while pre-clinical studies support the use of *ATRA* in the treatment of breast cancer (14–17). Finally, a number of pre-clinical and clinical studies provide insights into the therapeutic potential of *ATRA* in the context of *gastric cancer* (18–20).

Retinoid receptors, which are ligand-dependent transcription factors belonging to the family of nuclear steroid receptors (14, 21, 22), are the major mediators of the biological and pharmacological effects exerted by ATRA. Two classes of retinoid receptors are known, *Retinoic-Acid-Receptor*s (*RARα*, *RARβ*, *RARγ*) and *Retinoid-X-Receptor*s (*RXRα*, *RXRβ*, *RXRγ*), which act under the form of heterodimeric *RAR*/*RXR* complexes. In these complexes, *RAR*s function as the ligand-binding moiety (14). *RAR*/*RXR* complexes are involved in the modulation of gene-transcription and regulate the expression of numerous target genes. ATRA binds and activates all the *RAR*/*RXR* isoforms with similar affinity and efficiency. In addition, selective *RAR*/*RXR* synthetic agonists, such as AM580 (*RARα* agonist), CD2314 (*RARβ* agonist), CD4317 (*RARγ* agonist) and Bexarotene (RXR agonist), as well as antagonists, like BMS195614 (*RARα* antagonist), CD2665 (*RARβ/γ* antagonist) and LG100754 (RXR antagonist), are available (14).

In this single-institution/retrospective clinical and translational study (*RAR-GASTRIC*), we define the expression of *RARα*, *RARβ*, *RARγ*, *RXRα*, *RXRβ* and *RXRγ* in stomach tumors with the use of archival tissue samples obtained from *gastric cancer* patients. We investigate the associations between each *RAR*/*RXR* isotype and the clinical/biological characteristics of *gastric cancer*. In addition, we provide information regarding the associations between the *RAR*/*RXR* receptors and a simplified molecular classification derived from the *TCGA* (*The*-*Cancer*-*Genome*-*Atlas*) as well as the *ACRG* (*Asian*-Cancer-*Research*-*Group*) databases. Finally, we define the potential prognostic impact of *RAR*s and *RXR*s on the *overall*-*survival* of *gastric cancer* patients. The work supports the idea that *RARα*, *RARβ*, *RARγ* and *RXRβ* represent potential prognostic markers and actionable therapeutic targets in the context of the personalized treatment of *gastric cancer*.

## Materials and methods

### Study design

*RAR-GASTRIC* is a single-institution/retrospective clinical and translational study. In *RAR-GASTRIC*, we retrospectively collected and analyzed all the consecutive *gastric cancer* patients with archival tissue specimens available in the Pathology Unit of the *IRCCS CRO National Cancer Institute of Aviano* with confirmed diagnoses (from January 1, 2010 to March 30, 2019). We retrieved the data from electronic and paper-based chart reviews according to strict privacy standards. All the patients included in the study signed an anonymous *GE.CO* (*GESTIONE.CONTROLLO*) consent for the use of clinical data in the context of clinical research, epidemiology, training and disease-induction/-progression studies. The Departmental Review Board and the internal Ethics Committee (Protocol number CRO-2019-74) approved the *RAR-GASTRIC* study. The study was conducted in accordance with the Declaration of Helsinki, and approved by the Departmental Review Board and by the Ethics Committee of CRO Oncology Reference Center of Aviano, Italy (Protocol number CRO-2019-74). All included patients have signed a consent for the use of clinical data with the purpose of retrospective analyses aimed at clinical research. Informed consent was obtained from all subjects involved in the study.

### Objectives of the study

The primary objective of the *RAR-GASTRIC* study was to elucidate the expression and distribution of *RAR*s and *RXR*s in the general population of *gastric cancer* patients. The secondary objectives of the study were: 1) to determine associations between the expression of the various *RAR*/*RXR* mRNAs and clinical factors, such as stage, anatomical site and simplified molecular classifiers, which are available in the *TCGA* and *ACRG* datasets; 2) to characterize the prognostic impact of *RAR*s/*RXR*s expression on *overall*-*survival*.

### Sample preparation and RNA-expression analysis

Paraffin embedded tissues (core biopsies and gastrectomy samples) were sliced and reviewed by an expert pathologist. We extracted total RNA from the primary tumor samples obtained from biopsies or after surgery at diagnosis, using the *RNeasy DSPE FFPE* kit (Qiagen) and we assessed RNA quality using the *High Sensitivity RNA ScreenTape* kit (Agilent) and the *2200 TapeStation* system (Agilent). We performed RNA quantification with the *Qubit™ RNA HS* assay (Invitrogen) using the *Qubit fluorometer 2.0.* (Invitrogen). For each sample, we hybridized RNA (250 ng) with a customized panel of gene-probes (see below). We performed RNA detection and scanning using the *NanoString nCounter Analysis System* (NanoString Technologies) according to the manufacturer’s instructions. The background level of each tumor sample was obtained by subtraction of the Mean ± 2SD of the counts of the negative control probes included in the assay. We normalized the data using the geometric mean of the positive controls and the housekeeping genes (*B2M*, *GAPDH*, *HPRT1* and *RPL19*) included in the custom panel. We performed data analyses using the *nSolver 4.0* software (NanoString Technologies). We employed the Log_2_ transformed mRNA expression values for the analyses. The genes represented in our customized panel were: *CDH1* (*Cadherin-1*), *CDKN1A* (*Cyclin-Dependent-Kinase-Inhibitor-1A*), *EBER1* (*Epstein–Barr-Virus–Encoded-small-RNA1*), *MDM2* (*MDM2-Proto-Oncogene*), *MLH1* (*MutL-Homolog-1*), *MSH2* (*MutS-Homolog-2*), *MSH6* (*MutS-Homolog-6*), *PIK3CA* (*Phosphatidylinositol-4,5-Bisphosphate-3-Kinase-Catalytic-Subunit-Alpha*), *PMS2* (*PMS1-Homolog-2, Mismatch-Repair-System-Component*), *RARα* (*Retinoic*-*Acid*-*Receptor*-*Alpha*), *RARβ* (*Retinoic*-*Acid*-*Receptor*-*Beta*), *RARγ* (*Retinoic*-*Acid*-*Receptor*-*Gamma*), *RXRα* (*Retinoid*-*X-Receptor*-*Alpha*), *RXRβ* (*Retinoid*-*X-Receptor*-*Beta*), *RXRγ* (*Retinoid*-*X-Receptor*-*Gamma*), *TP53* (*Tumor*-*Protein*-*P53*) and *ZEB1 (Zinc-Finger-E-Box-Binding Homeobox1).* The normalization genes were: *B2M* (*Beta-2-Microglobulin*), *GAPDH* (*Glyceraldehyde-3-Phosphate Dehydrogenase*), *HPRT1* (*Hypoxanthine-Phosphoribosyltransferase-1*) and *RPL19* (*Ribosomal-Protein-L19*).

### Associations between overall-survival and gastric cancer patients’ clinical characteristics

We collected the data on sex, age, histology type, primary tumor site, stage at diagnosis, primitive-tumor resection, date of diagnosis of the metastatic disease, types and lines of treatment received (**Supplementary Table S1**). For the calculation of the *overall*-*survival* values, we used the time between the start of the treatment and death for any cause. For the calculation of the *overall*-*survival-after*-*metastatic*-*disease* values, we calculated the time between diagnosis of the metastatic disease and death for any cause. Given the observational nature of the study, we did not calculate any formal sample size. We summarized the patients’ clinical-pathological characteristics via descriptive analysis. We defined categorical variables on the basis of the frequency distribution, while we described continuous variables using the Mean ± SD, the Median of the first and third quartile (Q1-Q3) and the minimal as well as maximal values. We performed *Chi-square* or *Fisher’s* exact test, as appropriate, and *Kruskal*-*Wallis* tests to compare the distributions of the categorical and continuous variables, respectively. We explored the potential effects of the various *RAR*s and *RXR*s on *overall*-*survival* and *overall*-*survival-after*-*metastatic*-*disease* with the use of *COX* proportional hazard models, which we adjusted for the demographic and clinical prognostic characteristics of the patients. The results of these analyses were expressed as *Hazard-Ratios* (*HR*s) and 95% *Confidence-Intervals* (95%-*CI*s). We estimated the survival curves according to the *Kaplan*-*Meier* method and we compared them using the log-rank test. For bilateral tests, statistical significance was set at p<0.05. We performed subgroup analyses according to the clinical-pathological features and the molecular classifications. We performed statistical analyses using *STATA* (StataCorp. [2015] Stata Statistical Software: Release 14.2. College Station, TX: StataCorp LP).

## Results

### The cohort of gastric cancer patients: demographic characteristics

The total population of *gastric cancer* patients considered in the *RAR-GASTRIC* study consisted of 55 individuals (**Table 1**; **Supplementary Table S1**). To simplify the analysis of our data, we set a threshold age value of 70 to divide our population into two groups. In fact, this age is currently considered the standard threshold value above which aged Italian individuals are separated from the remainder of the Italian population. Forty-two patients (76%) were aged below 70 years, while 13 patients (24%) were aged above 70 years. Thirty-five patients (64%) were males, while 20 patients (36%) were females. Approximately one third of the primary stomach tumors localized into the cardias (33%) or in the corpus (31%), while the remaining cases localized into the antrum (18%) and fundus (9%), respectively. Thirty-nine patients (71%) underwent gastrectomy. At diagnosis, 18% of the cases showed early-stage tumors (*stage-I*/*stage-II*). In contrast, 38% and 33% of the patients suffered from locally advanced (*stage-III*) and advanced (*stage-IV*) tumors, respectively. Significantly, 18 cases (33%) suffered from *de novo* metastatic *gastric cancer*. Finally, 21 of the 39 individuals (38%), who underwent gastrectomy, experienced a relapse.

**Table 1:** Demographics of gastric cancer patients.

	No. patients	%		No. patients	%
**Age** (years)			**Distant recurrence**		
¾70	42	76	No	32	58
>70	13	24	Yes	21	38
**Gender**			**Lauren’s type**		
Male	35	64	Diffuse	30	55
Female	20	36	Intestinal	4	7
**Site at diagnosis**			Missing	21	38
Antrum	10	18	***HER2***		
Cardias	18	33	Negative	30	55
Corpus	17	31	Positive	4	7
Fundus	5	9	Missing	21	38
Missing	5	9	**Neoadjuvant therapy**		
**Gastric surgery**			No	36	66
Yes	39	71	Yes	19	34
**Stage at diagnosis**			**Adjuvant therapy**		
I-II	10	18	No	42	76
III	21	38	Yes	13	24
IV	18	33	**First line therapy**		
Missing	6	11	No	19	35
***De novo* metastatic disease**			Yes	29	53
No	32	58	Missing	7	12
Yes	18	33			
Missing	5	9			

The Table summarizes the clinical/pathological characteristics and the treatment protocols of the patients considered in the study.

**Figure 1:**
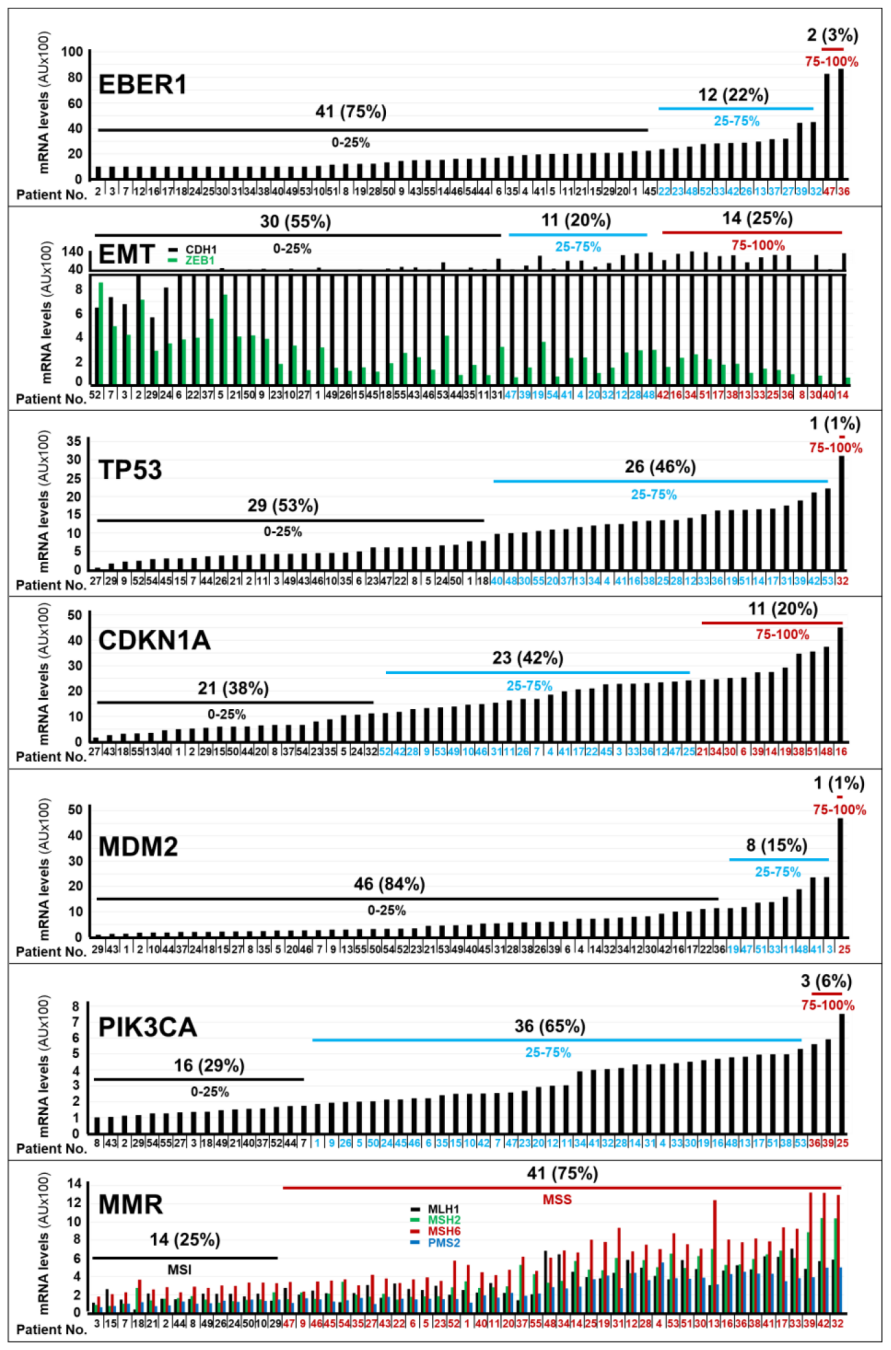
Expression levels of the mRNAs coding for different marker proteins in gastric cancer specimens. The bar-graphs illustrate the expression levels of the indicated mRNAs in the tumor samples of each cohort patient, which were determined with the use of the *NanoString nCounter Analysis System*. The genes considered are involved in *EBV* (*Epstein-Barr-Virus*) positivity (*EBER1*), in *EMT* (*Epithelial to Mesenchymal Transition*; *CDH1*/*ZEB1*), in the control of the *TP53* pathway (*TP53*/*CDKN1A*/*MDM2*), in the molecular landscape of *gastric cancer* (*PIK3CA*) and in the control of the *MMR* (*Mutation-Mismatch-Repair*; *MLH1*/*MSH2*/*MSH6*/*PMS2*) process. The number and percentage of patients (black values) presenting with the indicated expression quartiles (black blue and red values) of the various mRNAs considered. *We determined the normalized expression-levels (see Materials and Methods) of the indicated mRNAs in tumor tissues and we calculated the expression-level percentiles. We divided the calculated expression-level percentiles in tertiles, as indicated. Normalization mRNAs = *B2M* (*Beta-2-Microglobulin*); *GAPDH* (*Glyceraldehyde-3-Phosphate Dehydrogenase*); *HPRT1* (*Hypoxanthine-Phosphoribosyltransferase-1*); *RPL19* (*Ribosomal-Protein-L19*). Acronyms of the mRNAs considered: CDH1, Cadherin-1; ZEB1, Zinc-Finger-E-Box-Binding Homeobox1; TP53 = Tumor-Protein-P53, CDKN1A, Cyclin-Dependent-Kinase-Inhibitor-1A; EBER1, Epstein–Barr-Virus–Encoded-small-RNA1; MDM2, MDM2-Proto-Oncogene; MLH1, MutL-Homolog-1; MSH2, MutS-Homolog-2; MSH6, MutS-Homolog-6; PIK3CA, Phosphatidylinositol-4,5-Bisphosphate-3-Kinase-Catalytic-Subunit-Alpha; PMS2, PMS1-Homolog-2, Mismatch-Repair-System-Component; MSI, MicroSatellite-Instability; MSS, MicroSatellite-Stability.

As for the pathological characteristics (**Table 1**), the prevalent type of *gastric cancer* observed in our cohort of patients for whom data were available, was the *Lauren’s Diffuse* type (30 individuals = 55%). By converse, only a small fraction of patients (4 individuals; 7%) showed a *Lauren’s Intestinal* type of *gastric cancer*. In addition, a *HER2*-positive phenotype was evident in 18% of the *gastric cancer* cases. Finally, 25% of the tumor specimens presented with an *MSI* (*MicroSatellite-Instability*) status, due to a defective *MMR* (*DNA-Mismatch-Repair*) process, as indicated by the gene expression data (see **Figure 1**, lowest panel).

In terms of treatment (**Table 1**), approximately one third (18/55; 33%) and one quarter (13/55; 24%) of the patients were subjected to neo-adjuvant and adjuvant therapy, respectively. Data on first-line treatment were available for 48 patients and they indicated that 29 of the cases (53%) underwent this type of therapeutic strategy. Further details on the specific treatment(s) applied to each patient are available in **Supplementary Table S1**.

### Molecular characteristics and expression levels of the Retinoic Acid Receptors in gastric cancer

We approximated the *RAR-GASTRIC* set of tumor specimens to the molecular classification of *gastric cancer* available in the *TCGA* (*The-Cancer-Genome-Atlas*) and *ACRG* (*Asian-Cancer-Research-Group*) datasets (3, 4), by determining the RNA-expression levels of the 17 probes under study, which were contained in our customized panel of genes (*Nanostring* technology, see MATERIALS AND METHODS). The tumor tissues of only 2 patients (3%) showed high levels of the *EBER1* mRNA, a transcript coding for an *EBV* (*Epstein-Barr-Virus*) marker (**Figure 1**). This indicated that *EBV*-negative stomach tumors affected the vast majority of our patients. In addition, *gastric cancer* samples from 14 patients (25%) presented with a transcriptomic profile (low levels of *CDH1*; high levels of *ZEB1*) consistent with the presence of an *EMT* (*Epithelial-to-Mesenchymal-Transition*) phenotype (**Figure 1**). Only one patient expressed high levels of the *TP53* mRNA (**Figure 1**). With respect to the *TP53* pathway, it is interesting to notice that we found high expression levels of the *CDKN1A* mRNA in the tumor samples of 11 patients (20%) and large amounts of the *MDM2* (p53-binding protein) mRNA in a single patient (**Figure 1**). This information is relevant, as the *CDKN1A* and *MDM2* genes code for proteins involved in the control of p53 levels and activity. As for other genes of interest in the molecular landscape of *gastric cancer*, the neoplastic tissues of 3 patients (6%) were characterized by high levels of the *PIK3CA* mRNA. Finally, the expression profiles of genes involved in the *Mutation-Mismatch-Repair* (*MMR*) system (*MLH1*/*MSH2*/*MSH6*/*PMS2*) indicated that the vast majority of *gastric cancer* samples (41; 75%) showed a *Micro-Satellite-Stability* (*MSS*) phenotype. By converse, only a minority (14; 25%) of stomach tumors presented with a *Micro-Satellite-Instability* (*MSI*) phenotype (**Figure 1**).

**Figure 2:**
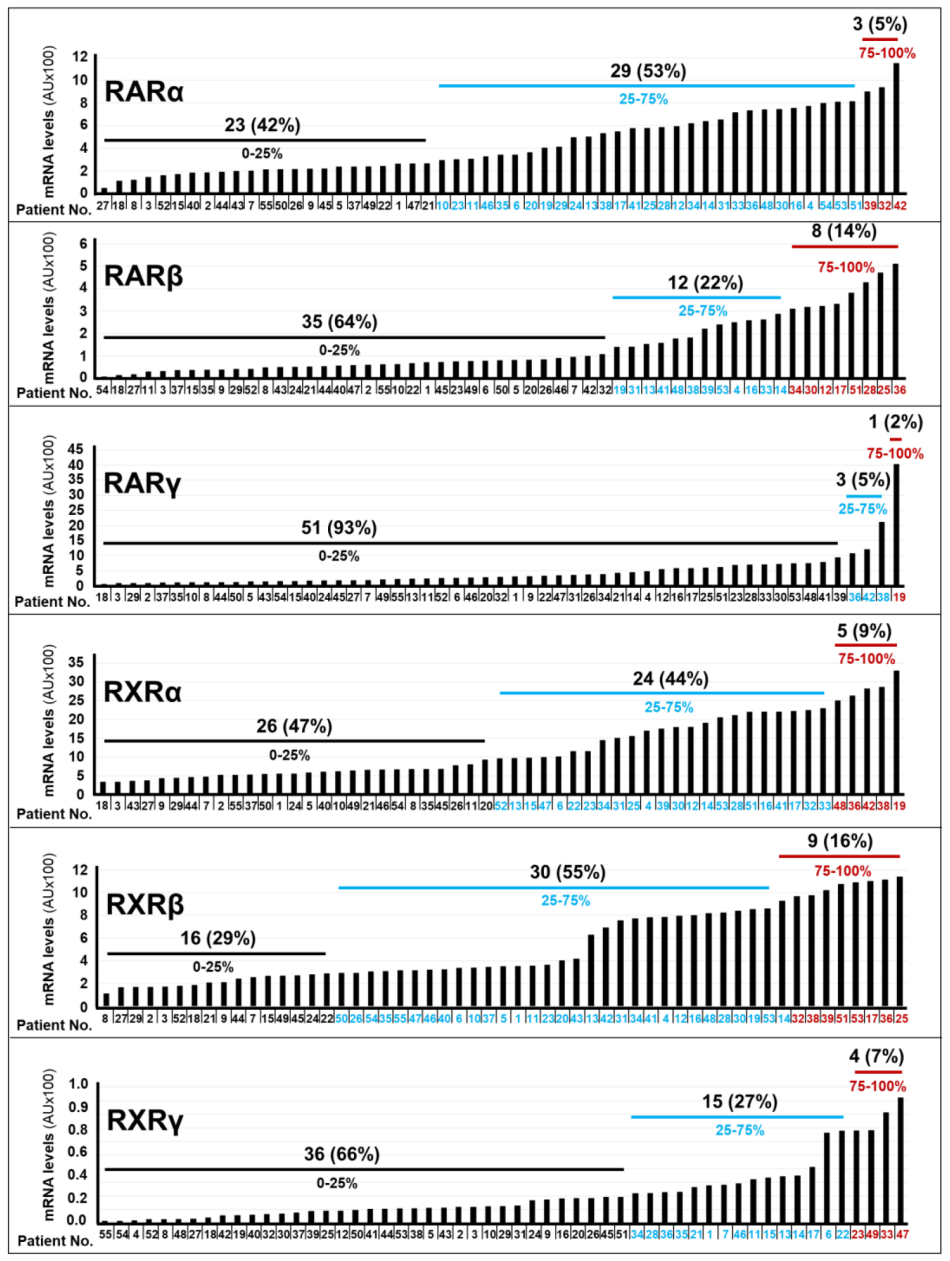
RAR/RXR mRNA levels in gastric cancer specimens. The bar-graphs illustrate the expression levels of the indicated RAR/RXR mRNAs in the tumor samples of each cohort patient, which were determined with the use of the *NanoString nCounter Analysis System*. The number and percentage of patients (black values) presenting with the indicated quartile (black blue and red values) of *RARα*, *RARβ*, *RARγ*, *RXRα*, *RXRβ* and *RXRγ* mRNA levels.

In our cohort of 55 patients, we determined the relative expression levels of the mRNAs coding for the *RARα*, *RARβ*, *RARγ*, *RXRα*, *RXRβ* and *RXRγ* receptors, with the use of our *Nanostring* data, (**Figure 2**). To perform this analysis, we measured the “*Absolute-Amount*” ranges of each receptor mRNA in our panel of tumor samples. The relative expression levels of the *RARα*, *RARβ*, *RARγ*, *RXRα*, *RXRβ* and *RXRγ* mRNAs were classified in 3 groups: “*High*” (75-100% maximal value), “*Intermediate*” (25-75% maximal value) and “*Low*” (0-25% maximal value). Following this type of analysis, 5%, 53% and 42% of the tumor samples showed high, intermediate and low expression levels of *RARα*, respectively. High levels of *RARβ* mRNA were evident in 14% of the specimens. By converse, 22% of the tumors presented with intermediate levels of the *RARβ* mRNA and we observed low levels of the transcript in 64% of the neoplastic tissues. In general, *RARγ* expression was deficient, as 93% of the patients presented with tumors characterized by low relative levels of this receptor isoform. A minority of cases (9%) expressed high levels of the *RXRα* transcript, while a large proportion of tumors showed intermediate (44%) or low (47%) levels of this mRNA. Finally, we observed intermediate amounts of the *RXRβ* mRNA in most of our samples (55%), whereas the *RXRγ* transcript showed a tendency towards low expression levels (66% of the cases). At present the molecular mechanisms underlying the selective upregulation of certain *RAR*/*RXR* isotypes in different subgroups of gastric cancer are unknown and further studies will be necessary to clarify the point.

### RARs/RXRs associations with gastric cancer clinical and molecular characteristics

**Figure 3:**
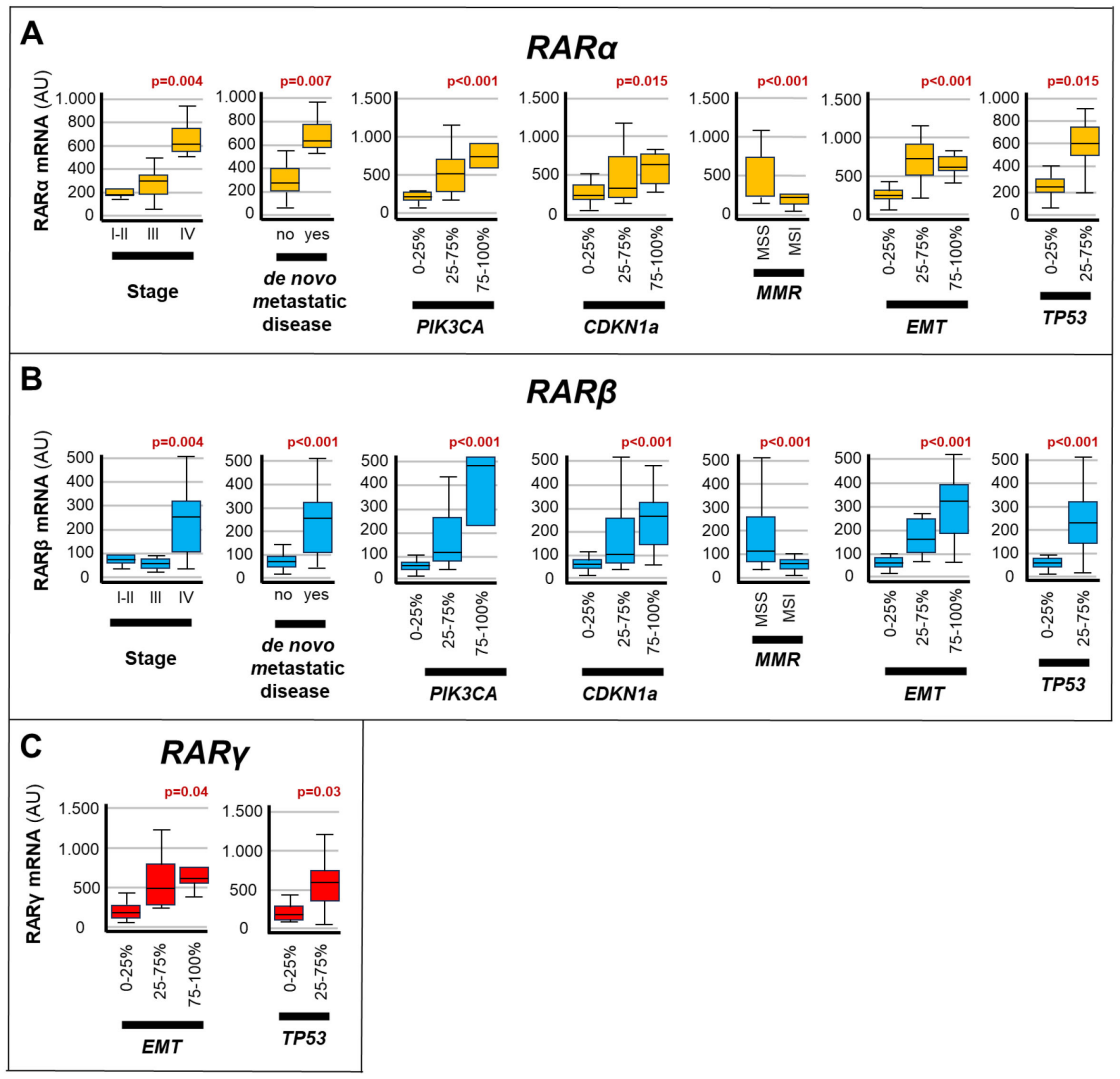
Associations between RARs levels and the clinical/molecular characteristics of gastric cancers. The box plots show the relative levels of the *RARα* **(A)**, *RARβ* **(B)** and *RARγ* **(C)** mRNAs in the indicated subgroups of *gastric cancers*. The relative levels of the 3 transcripts are grouped into expression quartiles, as indicated. The statistical-significance p-values of the correlations between the calculated *RARα*/*RARβ*/*RARγ* mRNA levels and the indicated clinical/molecular characteristics of the *gastric cancer* samples are shown in red above the corresponding box plots. Each panel illustrates only the p-values of the comparisons reaching statistical significance. PIK3CA, Phosphatidylinositol-4,5-Bisphosphate-3-Kinase-Catalytic-Subunit-Alpha; CDKN1A, Cyclin-Dependent-Kinase-Inhibitor-1A; EMT, Epithelial-to-Mesenchymal-Transition; MMR, DNA-Mismatch-Repair; MSI, MicroSatellite-Instability; MSS, MicroSatellite-Stability; TP53, Tumor-Protein-P53.

**Figure 4:**
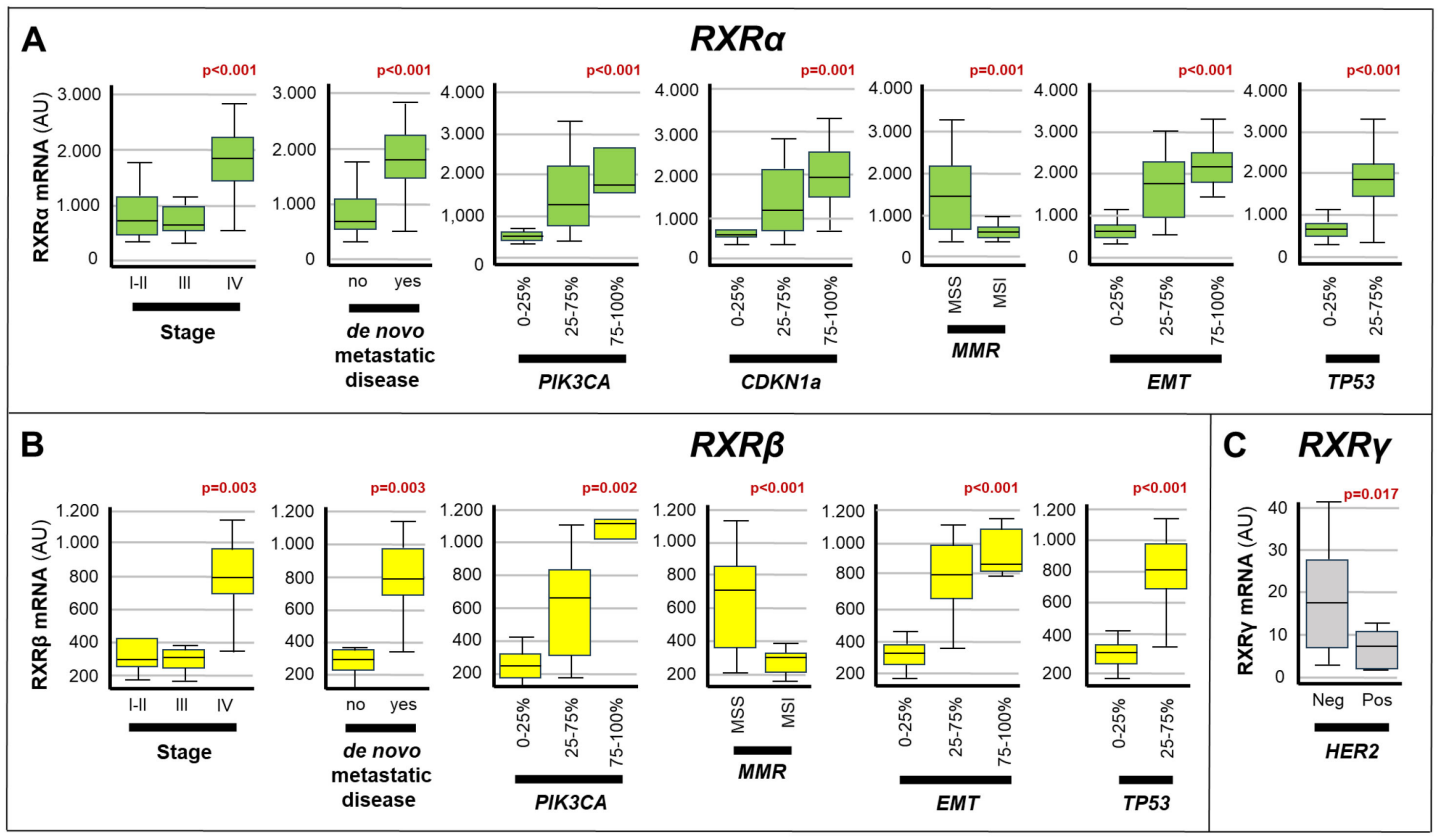
Associations between the expression of RXRs and the clinical/molecular characteristics of gastric cancers. The box plots show the relative levels of the *RXRα* **(A)**, *RXRβ* **(B)** and *RXRγ* **(C)** mRNAs in the indicated subgroups of gastric cancers. The statistical-significance p-values of the correlations between the calculated *RXRα*/*RXRβ*/*RXRγ* mRNA levels and the indicated clinical/molecular characteristics of the gastric cancer samples are shown in red above the corresponding box plots. Each panel illustrates only the p-values of the comparisons reaching statistical significance. PIK3CA, Phosphatidylinositol-4,5-Bisphosphate-3-Kinase-Catalytic-Subunit-Alpha; CDKN1A, Cyclin-Dependent-Kinase-Inhibitor-1A; EMT, Epithelial-to-Mesenchymal-Transition; MMR, DNA-Mismatch-Repair; MSI, MicroSatellite-Instability; MSS, MicroSatellite-Stability; TP53, Tumor-Protein-P53.

We investigated whether the expression levels of the various *RAR*/*RXR* isotypes (**Figures 3**, **4**) showed any statistical association with the clinical/molecular features of *gastric cancer*, which are listed in **Table 1**. As indicated in **Figure 3**, high levels of the *RARα* and *RARβ* mRNAs correlated with: stage-IV *gastric cancer* (*RARα*, p= 0.004; *RARβ*, p=0.004), *de novo* metastatic disease (*RARα*, p=0.007; *RARβ*, p<0.001), *MSS* (*RARα*, p<0.001; *RARβ*, p<0.001), *EMT* (*RARα*, p<0.001; *RARβ*, p<0.001) as well as high levels of the *PIK3CA* (*RARα*, p<0.001; *RARβ*, p<0.001), *CDKN1A* (*RARα*, p=0.015; *RARβ*, p<0.001) and *TP53* (*RARα*, p= 0.015; *RARβ*, p<0.001) mRNAs. By converse, high expression levels of the *RARγ* transcript presented significant associations only with *EMT* (p=0.04) and the content of *TP53* mRNA (p= 0.03).

As for the *RXR* transcripts (**Figure 4**), high expression levels of the *RXRα* mRNA showed significant correlations with: *stage-IV* disease (p<0.001), *de novo* metastatic disease (p<0.001), *MSS* status (p=0.001), *EMT* (p<0.001) as well as the amounts of the *TP53* (p< 0.001), *CDKN1A* (p=0.001) and *PIK3CA* (p<0.001) transcripts. In addition, the *RXRβ* mRNA moved in association with *stage-IV* disease (p=0.003), *de novo* metastatic disease (p=0.003), *MSS* status (p< 0.001), *EMT* (p<0.001) and high levels of *TP53* (p<0.001) as well as *PIK3CA* (p=0.002). Finally, high levels of the *RXRγ* transcript presented a significant correlation only with the *HER2*-negative status of *gastric cancer* (p=0.017).

### Associations of gastric cancer clinical and pathological factors with overall-survival

Based on a median follow-up of 50.8 months [*Inter-Quartile-range* (*IQr*) 22.1-93.6], the median *overall*-*survival* of our *gastric cancer* patients reached a value of 18.3 months (*IQr* 13.9-62.2), when this parameter was calculated from the diagnosis. If survival analysis was limited to the 37 patients experiencing a metastatic disease, a median *overall*-*survival* of 10.52 months (*IQr* 7.1-18.3) was calculated. Following univariate *COX*-regression analysis (**Table 2**), the main clinical/pathological factors showing significant correlations with a low *overall*-*survival* value were: *de novo* metastatic disease (p=0.001); distant recurrence (p=0.003); first-line therapy (p<0.001); *stage-IV* disease (p=0.01); high levels of the mRNAs involved in the *EMT* process (*CDH1* and *ZEB1*) (p=0.001) and high levels of *TP53* (p<0.001). In contrast, neo-adjuvant therapy and intermediate levels of the mRNAs involved in *EMT* (25%-75%) showed significant associations with a high *overall-survival* value. A last and relevant result of our analyses refers to the fact that neo-adjuvant therapy was also identified as a favorable prognostic factor (*HR*=0.44; 95%CI 0.20-0.95; p=0.04), being directly associated with high *overall*-*survival* (**Table 2**). Notably, the negative prognostic significance of *TP53* expression (p=0.008) and *stage-IV* disease (p=0.06, close to statistical significance) were substantially confirmed following multi-variate analysis (**Table 3**). The same type of multivariate analysis ratified the observation that intermediate expression levels of the mRNAs involved in *EMT* appeared as a favorable prognostic factor (p=0.02) (**Table 3**).

**Table 2:** Clinical-Molecular-Characteristics/overall-survival associations: univariate analysis.

	*HR (95% CI)*	*p-value*		*HR (95% CI)*	*p-value*
**Sex**			**Neoadjuvant therapy**		
Male	–	–	No	–	–
Female	1.67 (0.81-3.44)	0.16	Yes	0.44 (0.20-0.95)	0.04
**Age** (years)			**Adjuvant therapy**		
<70	–	–	No	–	–
>70	0.97 (0.44-2.15)	0.94	Yes	0.56 (0.21-1.47)	0.24
**Site at diagnosis**			***De novo* metastatic disease**		
Antrum	1.31 (0.50-3.44)	0.58	No	–	–
Cardias	–	–	Yes	3.37 (1.67-6.81)	0.001
Corpus	1.70 (0.74-3.88)	0.21	**Distant recurrence**		
Fundus	1.20 (0.26-1.54)	0.12	No	–	–
**Gastric Surgery**			Yes	2.79 (1.42-5.48)	0.003
Yes	–	–	**First line therapy**		
No	–	–	No	–	–
**cT stage**			Yes	6.95 (2.43-19.93)	p<0.001
I-II	–	–	***RAR/RXR***		
III-IV	1.44 (0.18-11.53)	0.73	*RARα*	1.99 (0.99-4.01)	0.053
**cN stage**			*RARβ*	2.95 (1.51-5.75)	0.001
N0	–	–	*RAR*γ	4.64 (1.51-5.75)	0.01
N positive	0.78 (0.15-11.53)	0.78	*RXRα*	1.60 (0.82-3.13)	0.17
**pT stage**			*RXRβ*	2.86 (1.25-6.55)	0.01
I-II	–	–	*RXR*γ	0.52 (0.25-1.09)	0.09
III-IV	2.29 (0.64-8.28)	0.21	***EBV***		
**pN**			0-25%	–	–
0	–	–	25-75%	1.61 (0.78-3.30)	0.2
1	1.16 e-16 (na)	0	75-100%	0.68 (0.09-5.09)	0.71
2	3.20 (0.81-12.70)	0.1	***EMT***		
3	2.38 (0.51-11.03)	0.27	0-25%	–	–
Missing	–	–	25-75%	2.31 (1.04-5.11)	0.04
**HER2-positivity**			75-100%	4.28 (1.84-9.95)	0.001
Negative	–	–	***TP53***		
Positive	1.25 (0.56-2.80)	0.58	0-25%	–	–
**Lauren’s type**			25-75%	3.67 (1.80-7.48)	<0.001
Intestinal	–	–	***CDKN1a***		
Diffuse	1.93 (0.25-14.75)	0.53	0-25%	–	–
**Stage at diagnosis**			25-75%	0.83 (0.39-1.75)	0.62
I-II	–	–	75-100%	0.98 (0.39-2.47)	0.97
III	1.36 (0.42-4.42)	0.61	***MDM2***		
IV	4.10 (1.36-12.37)	0.01	0-25%	–	–
***PIK3CA***			25-75%	0.50 (0.17-1.43)	0.2
0-25%	–	–	75-100%	4.10 e-15 (na)	1
25-75%	1.26 (0.60-2.64)	0.54	***MMR***		
75-100%	2.22 (0.48-10.33)	0.31	*MSS*	–	–
			*MSI*	0.61 (0.28-1.35)	0.22

The table illustrates the associations between the parameters marked in bold and the overall- survival values calculated for our patients. The table contains the calculated Hazard-Ratio (HR) values and the 95% confidence interval (95%-CI) values. For each variable of the univariate analysis, the statistical-significance p-value is indicated. When the calculated p-value reaches statistical significance (p<0.05), the results are underlined (inverse association) or marked in italics (direct association). na, not applicable.

**Table 3:** Clinical-Molecular-Characteristics/overall-survival associations: multivariate analysis.

	HR (95%-CI)	p-value
**Stage at diagnosis**		
I-II	–	–
III	1.38 (0.34-5.61)	0.45
IV	3.58 (0.96-13.36)	0.06
***RAR/RXR***		
*RARα*	0.63 (0.13-2.97)	0.56
*RARβ*	2.19 (0.28-17.10)	0.46
*RARγ*	4.28 (0.87-21.20)	0.08
*RXRβ*	1.61 (0.45-5.76)	0.46
***EMT***		
0-25%	–	–
25-75%	0.05 (0.004-0.58)	0.02
75-100%	0.12 (0.01-1.32)	0.08
***TP53***		
25-75%	11.01 (1.89-64.02)	0.008

The table illustrates a multi-variate analysis of the associations between the indicated clinical/ molecular characteristics or the levels of the indicated RAR/RXR mRNA and the overall- survival values calculated for our patients. The middle column contains the calculated Hazard-Ratio (HR) values and the 95% confidence interval (95%-CI) values. For each variable of the multivariate analysis, the statistical-significance p-value is shown in the rightmost column. When the calculated p-value reaches statistical significance (p<0.05), the results are underlined (inverse association) or marked in italics (direct association).

### Associations between retinoic acid receptors mRNA expression levels and overall-survival in gastric cancer

**Figure 5:**
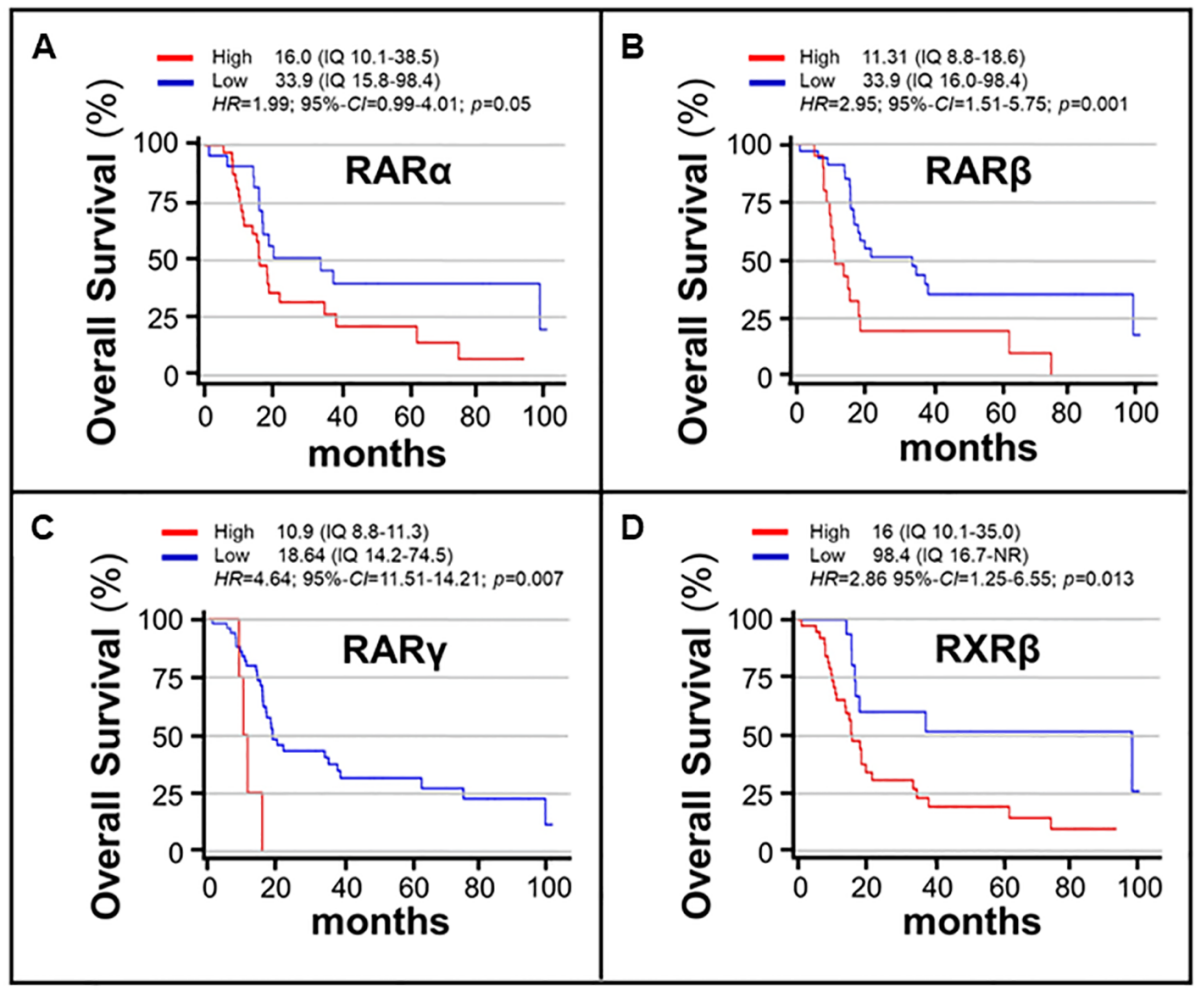
Associations between the overall-survival of gastric cancer patients and the expression levels of *RARα/RARβ/RARγ/RXRβ* mRNAs. The associations of the *RARα* **(A)**, *RARβ* **(B)**, *RARγ* **(C)** and *RXRβ* **(D)** mRNA expression levels and overall-survival values are illustrated by the curves. The expression levels of the various RAR and RXR isoforms are gathered binomially using the indicated expression ranges (IQ). The red curves illustrate the overall-survival rates of patients whose gastric cancer tumors are characterized by high levels of the indicated Retinoic-Acid-Receptor. The blue curves illustrate the overall-survival rates of patients whose gastric cancer tumors are characterized by low levels of the indicated Retinoic-Acid-Receptor. The associations were explored with the use of the COX proportional Hazard-Ratios (HRs). The analysis results are expressed as HRs and 95% Confidence-Intervals (95%-CIs). Survival curves are estimated according to the Kaplan-Meier method and compared using the log-rank test. Statistical significance is set at p<0.05 for bilateral tests.

In a last set of analyses, we determined possible associations between the expression levels of the various *RAR*/*RXR* mRNAs in our *gastric cancer* samples and *overall*-*survival*, using the same univariate *COX*-regression approach described in the previous chapter. The results obtained demonstrated that *RXRα* and *RXRγ* levels did not show any significant association with *overall*-*survival* (**Table 3**). In contrast, patients with high *RARα* expression levels were characterized by a significantly lower *overall*-*survival* relative to what was observed in patients endowed with small amounts of the *RARα* mRNA (16 months vs. 33.9 months; p= 0.05) (**Table 3**, **Figure 5A**). The same detrimental effect on *overall*-*survival* was observed in patients with high *RARβ* (p= 0.001) levels (**Table 3**, **Figure 5B**), high *RARγ* (p=0.007) levels (**Table 3**, **Figure 5C**) and high *RXRβ* (p= 0.013) levels (**Table 3**, **Figure 5D**). In the case of patients with high and low levels of *RARβ* expression, median *overall*-*survival* values of 11.31 months (*IQr* = 8.8-18-6) and 33.9 months (*IQr* = 16.0-98.4) were calculated. By the same token, patients with high levels of *RARγ* expression were associated with a poorer median *overall*-*survival* value (10.9 months; *IQr* = 8.8-11.3) than the low-expression counterparts (18.64 months; *IQr* = 14.2-74.5). The median *overall*-*survival* of *RXRβ* over-expressing tumors was 16.0 months (95%-*CI* = 10.1-35.0), as compared to 98.4 months in tumors showing low levels of the receptor (95%-*CI* = 16.7-NR) (**Figure 5**). Taken together, our data support the idea that the tumor levels of *RARα*, *RARβ*, *RARγ* and *RXRβ* mRNAs are potential determinants of a low *overall*-*survival* rate in *gastric cancer*.

## Discussion

*ATRA* is a vitamin-A metabolite, which is involved in cell and tissue maturation, particularly during embryonic development. The scientific literature supports the idea that pharmacologically active dosages of *ATRA* may be beneficial in the treatment and prevention of different types of solid tumors, including *gastric cancer* (11–20).

As already discussed in the Results section, the pharmacological activity of ATRA is mediated by the retinoic acid receptors, *RARα*, *RARβ*, *RARγ*, *RXRα*, *RXRβ* and *RXRγ*, which act under the form of *RAR*/*RXR* heterodimers. The development of ATRA-based therapeutic strategies in *gastric cancer* requires an initial assessment of the levels and relevance of *RAR*s/*RXR*s in this heterogeneous group of tumors. The present work is a retrospective archive-based translational study, whose major aim was to define the levels of the mRNAs encoding retinoic acid receptors in *gastric cancer*. The specific aims of the *RAR-GASTRIC* study were: a) to assess the *RAR*s/*RXR*s mRNA levels using archival *gastric cancer* specimens; b) to define potential associations between the expression levels of specific *RAR*s/*RXR*s and *gastric cancer* clinical features; c) to establish whether specific retinoic acid receptors were over-represented in any molecular subtype of *gastric cancer*, using our *TCGA*/*ACRG*-like classification; d) to assess the impact of *RAR*s and *RXR*s on the *overall*-*survival* of *gastric cancer* patients.

Our cohort consisted of 55 consecutive *gastric cancer* patients characterized by the presence of an archival histology specimen (biopsy or gastrectomy) which was collected during the time-period 2010-2019. The *RAR-GASTRIC* series contained a majority of male subjects (64%), and most of our patients (76%) fell under the age of 70 years. Interestingly, 66% of the patients presented with an initial diagnosis of localized disease. The overall-survival determined in our *gastric cancer* patients is in line with the data reported in clinical trials and the oncology literature (23), indicating that our cohort of patients was representative of the real-world population. In our cohort, we observed that 3% of the stomach tumors were *EBV*-positive, 25% of them were *EMT*-like cancers and 1% of the cases were *TP53*-positive. In addition, 20% and 25% of our *gastric cancer* cases classified as *HER2*-positive and *MSI* tumors, respectively. With respect to this last aspect, the scientific literature reports that *HER2*-positive and *MSI* tumors represent 15-30% and 10-30% of all stomach tumors, respectively (24).

The *RAR-GASTRIC* tumors were grouped according to our simplified version of the *TCGA*/*ACRG*-like classification of *gastric cancer*. At present, we cannot compare our analyses with a gold-standard validation procedure. In fact, the *TCGA*/*ACRG* molecular classification of *gastric cancer* lacks implementation in the clinical practice due to technical difficulties, costs, and the absence of direct therapeutic implications. Nevertheless, the prevalence of the molecular sub-groups in our cohort of patients and the *TCGA*/*ACRG* datasets is substantially overlapping in terms of *MSI* and *HER2* phenotypes, though slightly different in terms of *EBV*, *EMT* and *TP53* phenotypes.

As for the expression of the *RAR* and *RXR* transcripts, we confirmed that all these mRNAs were detectable in the *gastric cancer* samples, which we analyzed. In our tumor samples, we determined large amounts of the *RARα*, *RARβ*, *RXRα* and *RXRβ* transcripts. By converse, our data indicated that *gastric cancer* samples tended to express low levels of the *RARγ* and *RXRγ* mRNAs. The high levels of *RARα*, *RARβ* and *RXRα* determined in our tumor tissues associated with unfavorable clinical factors such as relapsed and “*de novo*” metastatic disease. This last observation supports the idea that advanced and high-stage stomach tumors contain large amounts of *RARα*, *RARβ*, *RXRα* and *RXRβ*. The determination of possible associations with the *gastric cancer* molecular features, which we examined, are substantially in line with the concept that *RARα*/*RARβ*/*RXRα*/*RXRβ* levels correlate with *MSS*, *EMT* and *TP53* expression in a positive manner. Thus, our data indicate that expression of these four retinoic acid receptors is linked to stomach tumors characterized by an aggressive molecular phenotype and poor overall-survival.

In the *RAR-GASTRIC* study, we also focused on possible associations between the *RAR*/*RXR* mRNA levels and the expression of the two transcripts encoding *PIK3CA*/*CDKN1A*. The *PIK3CA*-gene is of interest because about 15% of the stomach tumors present with a mutation observed predominantly in the *EBV*-positive molecular subgroup (4, 5, 25). Indeed, *PIK3CA* is a key determinant of various intra-cellular signaling pathways, as it is involved in processes such as cell proliferation and survival. In addition, mutations of the *PIK3CA* gene are potential pharmacological targets of aromatase-inhibitor resistant breast cancers (26, 27). Finally, there is pre-clinical evidence of an interplay between *ATRA* and *PIK3CA* in the control of cell proliferation (28, 29). As for the *CDKN1A*-gene, the encoded protein is an inhibitor of *CDK2*, a cyclin dependent kinase involved in the control of cell cycle. Moreover, the *TP53* tumor suppressor controls *CDKN1A* expression (https://www.ncbi.nlm.nih.gov/gene/1026). Finally, *TP53*, *PIK3CA* and *CDKN1A* are part of an integrated pathway controlling p53 functional activity, which identifies p53-active and -inactive tumors in a simple manner (5). Studies available in the scientific literature indicate that *ATRA* targets *CDK2*, causing an arrest of breast cancer cell proliferation (30). Other studies link ATRA to the *CDK*-mediated cell proliferation processes (31, 32). Interestingly, we demonstrated that the levels of *RARα*, *RARβ* and *RXRα* show a significant association with *TP53*, *PIK3CA* and *CDKN1A* expression. In addition, we observed that *RXRβ* expression correlated with *TP53* and *PIK3C* expression levels. Finally, we established associations of the *RARα*, *RARβ*, *RARγ* and *RXRβ* levels with a detrimental effect on the overall-survival of *gastric cancer* patients.

## Conclusions

The results of the *RAR-GASTRIC* study demonstrate that *gastric cancer* specimens contain significant amounts of *RARα*/*RARβ*/*RXRα*/*RXRβ* mRNAs. By converse, the levels of *RARγ* and *RXRγ* tend to be low. Based on our results, it is reasonable to assume that high expression levels of *RARα*, *RARβ*, *RXRα* and *RXRβ* have a negative impact on *gastric cancer* patients’ overall-survival. In fact, high levels of *RARα*, *RARβ*, *RXRα* and *RXRβ* are associated with unfavorable prognostic clinical and pathological characteristics of *gastric cancer*, such as stage-IV and “*de novo*” metastatic disease. In addition, there are *gastric cancer* molecular subtypes (*MSS*, *EMT*-like and possibly p53-inactive cancers), which are enriched in specific types of retinoic acid receptors. The relevance of our study resides in the originality of the concept and the attempt to link the evaluation of *ATRA* receptors expression, not only to clinical characteristics but also to a reproducible as well as simplified molecular classification of *gastric cancer* based on the use of a limited number of molecular-subtypes hallmarks.

Clearly, further studies are necessary to add robustness to the clinical/biological associations that we determined. Nevertheless, our results indicate that specific retinoic acid receptors represent potential *gastric cancer* prognostic markers. In addition, the data contained in this study have significant implications in the context of the stratified/personalized treatment of *gastric cancer*. In fact, they support the idea that certain retinoic acid receptors represent targets for the development of innovative therapeutic strategies based on *ATRA* or available *RAR*/*RXR* agonists/antagonists. Finally, association studies on RAR/RXR expression levels and treatment response are necessary and must be conducted on a larger cohort of patients.

## Data Availability

Original datasets are available in a publicly accessible repository: The original contributions presented in the study are publicly available. This data can be found here: https://github.com/silvioken/RAR-Gastric/blob/main/Repository.xlsx.

## Supplementary material

Supplementary Table 1 Overview of the treatments for each patient.
